# Associations of marrow fat fraction with MR imaging based trabecular bone microarchitecture in first-time diagnosed type 1 diabetes mellitus

**DOI:** 10.3389/fendo.2024.1287591

**Published:** 2024-05-07

**Authors:** Wei Li, Wei Wang, Minlan Zhang, Qi Chen, Shaojun Li

**Affiliations:** ^1^ Department of Radiology, Shanghai University of Medicine & Health Sciences Affiliated Zhoupu Hospital, Shanghai, China; ^2^ Department of Laboratory Medicine, Shanghai University of Medicine & Health Sciences Affiliated Zhoupu Hospital, Shanghai, China

**Keywords:** type 1 diabetes mellitus, marrow adiposity, magnetic resonance spectroscopy, trabecular microarchitecture, diabetes mellitus

## Abstract

**Purpose:**

To determine whether there are alterations in marrow fat content in individuals first-time diagnosed with type 1 diabetes mellitus (T1DM) and to explore the associations between marrow fat fraction and MRI-based findings in trabecular bone microarchitecture.

**Method:**

A case-control study was conducted, involving adults with first-time diagnosed T1DM (n=35) and age- and sex-matched healthy adults (n=46). Dual-energy X-ray absorptiometry and 3 Tesla-MRI of the proximal tibia were performed to assess trabecular microarchitecture and vertebral marrow fat fraction. Multiple linear regression analysis was used to test the associations of marrow fat fraction with trabecular microarchitecture and bone density while adjusting for potential confounding factors.

**Results:**

In individuals first-time diagnosed with T1DM, the marrow fat fraction was significantly higher (*p* < 0.001) compared to healthy controls. T1DM patients also exhibited higher trabecular separation [median (IQR): 2.19 (1.70, 2.68) vs 1.81 (1.62, 2.10), *p* < 0.001], lower trabecular volume [0.45 (0.30, 0.56) vs 0.53 (0.38, 0.60), *p* = 0.013], and lower trabecular number [0.37 (0.26, 0.44) vs 0.41 (0.32, 0.47), *p* = 0.020] compared to controls. However, bone density was similar between the two groups (*p* = 0.815). In individuals with T1DM, there was an inverse association between marrow fat fraction and trabecular volume (*r* = -0.69, *p* < 0.001) as well as trabecular number (*r* = -0.55, *p* < 0.001), and a positive association with trabecular separation (*r* = 0.75, *p* < 0.001). Marrow fat fraction was independently associated with total trabecular volume (standardized β = -0.21), trabecular number (β = -0.12), and trabecular separation (β = 0.57) of the proximal tibia after adjusting for various factors including age, gender, body mass index, physical activity, smoking status, alcohol consumption, blood glucose, plasma glycated hemoglobin, lipid profile, and bone turnover biomarkers.

**Conclusions:**

Individuals first-time diagnosed with T1DM experience expansion of marrow adiposity, and elevated marrow fat content is associated with MRI-based trabecular microstructure.

## 1 Introduction

Diabetic bone fragility is a complication of both type 1 and type 2 diabetes mellitus (T1DM, T2DM) and differs from senile and postmenopausal osteoporosis (1, 2). Marrow adipose tissue is a unique type of fat depot that differs from other fat depots in the body. It is sensitive to insulin and possesses distinct metabolic and molecular characteristics that can affect overall energy metabolism (3, 4). Expansion of marrow fat can occur as a result of aging or certain pathologies such as osteoporosis, anorexia nervosa, diabetes mellitus, impaired hematopoiesis, or deficiencies in estrogen and growth hormone (1, 5, 6). Generally, an increase in marrow fat is associated with decreased skeletal integrity (7–9). Since decreased osteoblastogenesis (the formation of new bone) has been observed in T1DM (10), investigating the content of marrow fat is worthwhile.

The phenotype (physical characteristics) of marrow adipose tissue is somewhat unclear. Studies in mouse models of T1DM consistently showed an increase in marrow fat content (6, 11–13). However, when assessing marrow fat content in T1DM patients, some unexpected results were found. Several studies showed no significant difference in marrow fat content between T1DM patients and controls at various skeletal sites such as the lumbar spine, tibia metaphysis, femur epiphysis, and femur metaphysis (14–17). These studies suggest that further clinical research is needed to fully understand whether marrow adipose tissue is altered in T1DM, particularly in first-time diagnosed cases.

Therefore, the purpose of this study was to determine whether there are changes in marrow fat content in first-time diagnosed T1DM patients and to investigate the relationships between marrow fat content and MRI-based findings of trabecular bone microarchitecture.

## 2 Methods

### 2.1 Subjects

In this cross-sectional study, we recruited thirty-five first-time diagnosed patients with T1DM from the endocrinology clinic at Shanghai University of Medicine & Health Sciences Affiliated Zhoupu Hospital, during the period spanning from August 2018 through May 2023. The main inclusion criteria was as follows: 1) age 18 years and older; 2) able to understand the study protocol; 3) diagnosis of diabetes based on the World Health Organization 1999 criteria. Exclusion criteria included: 1) chronic diseases that could interfere with the assessment of the variables, such as renal and hepatic chronic diseases; 2) individuals receiving medications affecting bone metabolism (i.e., glucocorticoids, hormone replacement therapy, bisphosphonates, denosumab, teriparatide, strontium ranelate, and calcitonin); 3) co-existing acute diseases, such as acute myocardial infarction and infection, that could affect glucose metabolism. We also recruited forty-six age-, gender-, and body mass index (BMI)-matched healthy controls from the community during the same period, and the exclusion criteria were the same as those for the T1DM cohort.

We collected various clinical data, including age, gender, BMI, personal health, medication history, and lifestyle habits, such as dairy intake, use of vitamins or calcium supplementation, cigarette smoking, and alcohol consumption. Physical activities were defined as engaging in exercise three times a week or more, with each session lasting at least 30 minutes. The study protocol was reviewed and approved by the Institutional Review Board of Shanghai University of Medicine & Health Sciences Affiliated Zhoupu Hospital. All participants provided informed consent prior to their participation in the study.

### 2.2 Biochemical analysis

Blood samples were collected between 8 and 9 a.m. after an overnight fast to minimize confounding factors and ensure accurate assessment of bone health. Enzymatic methods (ADVIA Chemistry XPT; SIEMENS) were used to measure serum creatinine, uric acid, calcium, phosphorus, serum lipid profile (including total cholesterol, high-density lipoprotein cholesterol [HDL-C), low-density lipoprotein cholesterol (LDL-C), and triglyceride (TG)], fasting blood glucose (FBG), and postprandial blood glucose (PBG). Electrochemiluminescence assays (Cobas e601; Roche Diagnostics, Basel, Switzerland) were used to determine serum 25-hydroxyvitamin D [25-(OH)D], total osteocalcin, intact N-terminal propeptide of type I collagen (PINP), and C-terminal crosslinked telopeptides of type I collagen (CTX). Plasma glycated haemoglobin (HbA1c) was determined using high performance liquid chromatography (MQ-6000; Shanghai Medconn Biotechnology Co., Ltd., China).

### 2.3 Dual-energy x-ray absorptiometry

The area bone mineral density (BMD, g/cm^2^) of the lumbar spine is an important measure of bone health and is commonly estimated using dual-energy X-ray absorptiometry (DXA). In our study, we utilized the Lunar Prodigy DXA machine manufactured by GE Healthcare in Milwaukee, WI, USA. This machine is widely used and is known for its accuracy and reliability in measuring BMD. In our center, we have established strict quality control measures to ensure the reliability of the DXA scan. One important measure of reliability is the coefficient of variation. In our study, the coefficient of for the lumbar spine BMD measurements was found to be 1.24%.

### 2.4 MRI assessment of microarchitecture

We captured high-resolution MRI images of the proximal tibia using a 3-T MRI scanner (MAGNETOM Skyra; Siemens Medical Systems, Erlangen, Germany) with a transmit/receive extremity coil suitable for knee imaging. To acquire trabecular images, we utilized a fully balanced steady-state free precession sequence with the following parameters: repetition time (TR) = 10.83 ms, echo time (TE) = 4.69 ms, matrix = 448 × 448, flip angle = 60°, number of averages = 15, number of slices = 20, field of view = 100 mm × 100 mm, and bandwidth (Hz) = 189.

The standardized analysis was performed using the slice located at the insertion point of the patellar ligament. The coded images were then blindly analyzed using MATLAB (MathWorks Inc., San Mateo, CA) to obtain measurements of trabecular parameters. We applied an advanced global thresholding method, using two reference intensity levels: one for marrow fat (Im) and the other for bone (Ib) in image segmentation. To establish the bone reference (Ib), we derived cortical bone intensity from smoothed intensity profiles perpendicular to the cortical bone surface. Cortical intensity for each ray was determined as the first minimum beyond the trabecular region, and the median value from 10 rays was chosen as the cortical intensity for a slice. Meanwhile, the marrow reference intensity (Im) was automatically determined by analyzing the intensity histogram within the trabecular bone region, following a previously established procedure (18, 19). The global threshold, based on the mean intensity of the trabecular region (Itrab), resulted in a bone fraction BV/TV = (Ttrab - Ib)/(Im - Ib). This method ensures the preservation of the average bone fraction within the area, particularly when intensity changes are solely attributed to the partial volume effects of bone and marrow.

The analysis focused on a specific region of trabecular bone in the proximal tibia. Using the segmented binary image, we counted bone pixels within each region of interest (ROI) and calculated the ROI area to determine the apparent bone volume fraction (App BV/TV). To quantify the total number of edges (IL) between bone and marrow phases, we projected rays through the 3D image for each angle pair (θ, φ), with φ set to 0 for 2D analysis. Then, we calculated the mean intercept length (MIL) as L(θ, φ) by multiplying 2 times the App BV/TV with the total length (TL) of lines traversing the image and dividing by IL. Subsequently, we computed the 3D fabric tensor, H, using mean intercept lengths and robust least squares fitting. Eigenvalues were derived through singular value decomposition, with MIL1 > MIL2 > MIL3 representing mean trabecular thickness along primary orientations. The largest eigenvalue and its orientation vividly indicated the main trabecular orientation. Conversely, the other eigenvalues suggested less favored orientations, while under isotropic conditions, all eigenvalues would be comparable. We analyzed structural anisotropy by computing eigenvalue ratios: MIL1/MIL3, MIL2/MIL3, and MIL1/MIL2. Using a slice-by-slice method, we also determined apparent trabecular thickness [appTbTh (mm)], apparent trabecular separation [appTbSp (mm)], and apparent trabecular number [appTbN (1/mm)]. Precision errors were assessed through three scans and analyses on three participants, with repositioning between each session. Subsequently, we calculated the root-mean-square (RMS) average coefficient of variation (CV%). Reproducibility errors, as indicated by the RMS average CV%, ranged from 1.23% to 3.85% across trabecular bone microstructure measurements.

The study involved two musculoskeletal radiologists, L.W. and L.S., who had extensive experience in musculoskeletal MRI, with L.W. having more than 5 years and L.S. having more than 10 years of experience. Both radiologists independently measured trabecular parameters without knowledge of the clinical findings, ensuring unbiased assessments. To evaluate the consistency of the measurements, Two-way, random-effects model, single-measure intraclass correlation coefficients (ICCs) were employed, which accounted for both intra- and inter-observer variability. The strength of the ICC was categorized as follows: 0–0.39 indicating poor agreement, 0.40–0.59 indicating fair agreement, 0.60–0.74 indicating good agreement, and 0.75–1.0 indicating excellent agreement (20).

### 2.5 MR spectroscopy

In addition, the researchers conducted MR spectroscopy to determine the amount of fat present in the vertebral marrow. The participants were positioned in a supine position inside the scanner. To meet the specific clinical needs of each individual, a standard clinical MRI of the lumbar spine was performed. This included sagittal turbo-spin echo T1- and T2-weighted imaging, as well as transaxial T2-weighted fast spin echo sequence. Following this, a stimulated echo acquisition method (STEAM) sequence was used to measure the percentage of bone marrow fat using MR spectroscopy.

**Figure 1:**
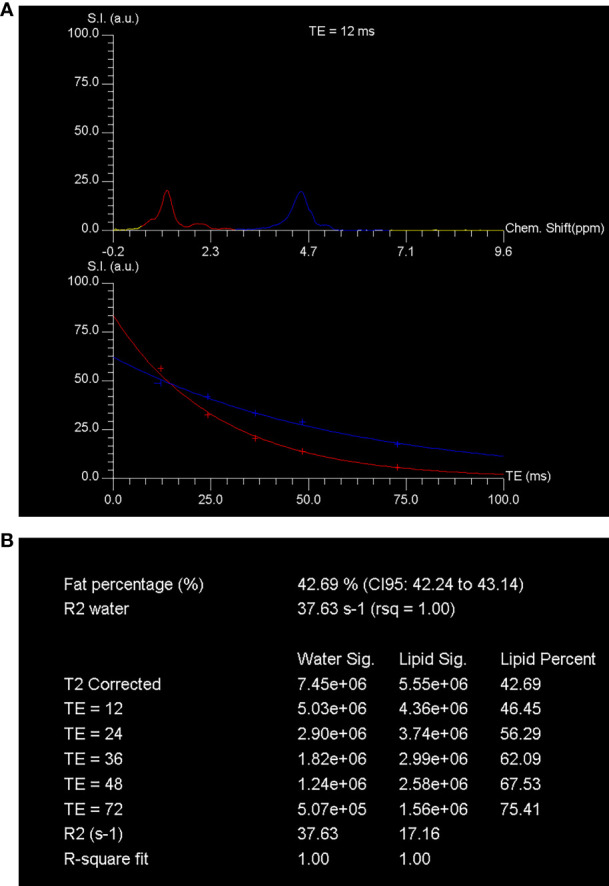
MR spectroscopy assessment of the fat content in vertebral marrow. The spectroscopy was performed using STEAM (stimulated echo acquisition mode) techniques. The technique involved acquiring single-voxel T2-weighted proton spectra at different echo times (TEs) of 12, 24, 36, 48, and 72 ms, along with a repetition time (TR) of 3000 ms. The spectra obtained at a TE of 12 ms showed distinct peaks corresponding to water and fat. The water peak appeared at 4.7 ppm, while the fat peak appeared at 1.30 ppm. These peaks were represented by blue and red curves, respectively, in **(A)**. The signal intensity of these peaks was quantified at each TE. To determine the fraction of marrow fat, the researchers calculated the percent of marrow fat fraction using the T2-corrected fat and water signal intensities. The calculated fraction was found to be 42.7% **(B)**. This calculation was based on the assumption that the fat and water signals followed a T2 decay curve. Chem., chemical; CI, confidence interval; rsq, r-squared; S.I. (a.u.), signal intensity (arbitrary units).

For lumbar MR spectroscopy, a voxel measuring 1.5 cm × 1.5 cm × 1.5 cm was placed in the L3 vertebral body. The volume of interest was positioned in the middle of the vertebral body, and the size of the voxel remained constant throughout the study. The STEAM pulse sequence was used to collect data for single-voxel MR spectroscopy without water suppression. The parameters for the sequence were as follows: TR = 3000 ms, TEs = 12, 24, 36, 48, and 72 ms, data points = 1024, receiver bandwidth = 2000 Hz. Gradient shimming and optimization of transmit and receive gain were performed using automated procedures. The signal intensities of the fat and water peaks were measured at each echo time by integrating the corresponding spectral region. After adjusting for T2 decay by fitting an exponential function to the signal change with echo time, the percentage of fat was calculated using the signal intensities of fat and water (21), as illustrated in **Figure 1**.

### 2.6 Statistical analysis

The descriptive statistics included in the analysis were the mean ± standard deviation (SD), median (25th – 75th percentile), counts, and frequencies. The normality of the data was assessed using the Shapiro-Wilks test. Continuous data were compared using Student’s t-test or Mann-Whitney U test, depending on the distribution of the data. Categorical data were compared using the χ2 test or Fisher’s exact test, as appropriate. The association between variables was assessed using Pearson’s or Spearman’s test. Multiple linear regression was performed to examine the associations of MRI-based trabecular microarchitecture with marrow fat fraction, while adjusting for various covariates such as age, gender, body mass index, physical activity, smoking status, alcohol consumption, blood glucose, plasma glycated hemoglobin, lipid profile, and bone turnover biomarkers. Statistical analysis was conducted using SPSS version 25, and a *p*-value less than 0.05 was considered statistically significant.

## 3 Results

### 3.1 Clinical characteristics and biochemical markers of bone metabolism

**Table 1** presents the characteristics of the study population, including both the first-time diagnosed T1DM patients and the controls. There were no significant differences in demographics or anthropometric measurements between the two groups. However, the levels of triglyceride were found to be higher in the adults with first-time diagnosed T1DM compared to the controls. When comparing markers of bone metabolism, including plasma 25-hydroxyvitamin D, PINP, total osteocalcin, and CTX levels, there were no significant differences between the T1DM group and the control group (**Table 1**).

**Table 1:** Descriptive characteristics of T1DM and controls included in the study.

Parameters	T1DM (n=35)	Controls (n=46)
Age (years), median (IQR)	33.5 (23.0, 42.3)	34.0 (23.0, 45.5)
Female gender	14 (40%)	13 (37%)
BMI (kg/m^2^)	21.5 (19.0, 23.7)	21.8 (19.5, 23.8)
Current smoking, n (%)	10 (29%)	9 (26%)
Alcohol drinkers, n (%)	5 (14%)	4 (11%)
Physical activity, n (%)	12 (34%)	14 (40%)
FBG (mmol/L)	9.1 ± 2.2	4.7 ± 1.1 *
PBG (mmol/L)	16.7 ± 5.3	5.9 ± 1.4 *
HbA1C (%)	9.5 ± 2.8	5.1 ± 0.6 *
Total cholesterol (mmol/L)	4.65 ± 1.0	4.41 ± 0.91
Triglycerides (mmol/L)	0.83 ± 0.30	0.55 ± 0.22*
HDL-C (mmol/L)	1.48 ± 0.46	1.65 ± 0.47
LDL-C (mmol/L)	2.52 ± 0.68	2.41 ± 0.58
25-hydroxyvitamin D (ng/ml)	17.6 (10.4, 23.9)	18.5 (13.6, 28.1)
PINP (ng/ml)	38.4 (25.9, 47.3)	37.3 (26.3, 47.1)
Osteocalcin (ng/ml)	15.3 (9.2, 21.6)	16.2 (9.0, 23.4)
CTX (ng/ml)	0.98 (0.63, 1.41)	0.95 (0.60, 1.44)

Data are presented as mean ± standard deviation, median (interquartile range), or n (%).

*P < 0.05 compared with the T1DM group. Comparisons were performed with chi-square test for categorical variables, and Mann-Whitney test or t test for continuous variables depending on the normal distribution.

BMI, body mass index; CTX, C-terminal crosslinked telopeptides of type I collagen; FBG, fasting blood glucose; HbA1c, hemoglobin A1c; HDL-C, high-density lipoprotein cholesterol; LDL-C, low-density lipoprotein cholesterol; PBG, postprandial blood glucose; PINP, intact N-terminal propeptide of type I collagen; T1DM, type 1 diabetes mellitus.

### 3.2 Inter- and intra-reader agreements

There was excellent inter-reader agreement for appBV/TV (ICC = 0.93; 95% CI: 0.90–0.97), appTbN (ICC = 0.91; 95% CI: 0.89–0.95), appTbSp (ICC = 0.93; 95% CI: 0.91–0.97) and appTbTh (ICC = 0.90; 95% CI: 0.87–0.94). Intra-reader agreements for measurements of MRI trabecular bone microarchitecture were likewise excellent, with all ICCs ranging between 0.91–0.97 (95% CI: 0.93–0.96).

### 3.3 BMD, MRI trabecular bone microarchitecture, and bone marrow adiposity between the cases and controls

Furthermore, the assessment of bone mineral density (BMD) of the lumbar spine using dual-energy X-ray absorptiometry showed no difference between the T1DM group and the control group (**Table 2**). However, when examining bone microarchitecture of the proximal tibia using MRI-based measures, it was found that trabecular bone microarchitecture variables differed between the T1DM group and the control group. Specifically, appTbSp was significantly higher in T1DM, while appBV/TV and appTbN were lower in T1DM compared to controls. The appTbTh was also lower in T1DM, but this difference did not reach statistical significance. Additionally, the mean vertebral marrow fat fraction was higher in T1DM cases than in controls.

**Table 2:** BMD, MRI trabecular bone microarchitecture and bone marrow adiposity between the two groups.

Variables	T1DM (n=35)	Controls (n=46)	*p*-values
Lumbar spine BMD (g/cm^2^)	1.120 ± 0.165	1.178 ± 0.137	0.816
appBV/TV (%)	0.45 (0.30, 0.56)	0.53 (0.38, 0.60)	0.013
appTbN (1/mm)	0.37 (0.26, 0.44)	0.41 (0.32, 0.47)	0.020
appTbSp (mm)	2.19 (1.70, 2.68)	1.81 (1.62, 2.10)	<0.001
appTbTh (mm)	1.02 (0.89, 1.27)	1.09 (0.90, 1.29)	0.900
Marrow fat fraction (%)	39.2 ± 5.8	35.3 ± 5.4	<0.001

Data are presented as mean ± standard deviation, median (interquartile range).

Comparisons were performed with Mann-Whitney test or t test for continuous variables depending on the normal distribution.

App, apparent; BV/TV, bone volume/total volume fraction; TbTh, trabecular thickness; TbN, trabecular number; TbSp, trabecular separation; BMD, bone mineral density.

### 3.4 Associations of marrow fat fraction with BMD and MRI trabecular bone microarchitecture

In the T1DM cases, a mild correlation was observed between vertebral marrow fat fraction and BMD at the lumbar spine (*r* = −0.40, *p* = 0.024). Moreover, marrow fat fraction values were found to be negatively correlated with appBV/TV (*r* = −0.69, *p* < 0.001) and appTbN (*r* = −0.55, *p* < 0.001), and positively correlated with appTbSp (*r* = 0.75, *p* < 0.001). However, no association was found between vertebral marrow fat fraction and appTbTh (*r* = −0.25, *p* = 0.127). To further analyze the relationship between marrow fat fraction, BMD, and MRI-based trabecular bone microarchitecture, a multivariate linear regression model was employed. The results were adjusted for various potential confounding factors, including age, gender, body mass index, physical activity, smoking status, alcohol consumption, blood glucose, plasma glycated haemoglobin, lipid profile, and biochemical markers of bone metabolism. The regression model showed that bone marrow fat fraction was independently associated with total bone volume (standardized β = −0.21), trabecular number (standardized β = −0.12), and trabecular separation (standardized β = 0.57). These associations remained significant after adjusting for confounding factors (*p* < 0.05 for all) (**Table 3**).

**Table 3:** Multiple linear regression analysis of MRI-based measures of trabecular bone microarchitecture and BMD on vertebral marrow adiposity in the T1DM group.

Variables	Standard β ± SE	*p*-values
appBV/TV (%)	− 0.21 ± 0.04	< 0.001
appTbN (1/mm)	− 0.12 ± 0.03	0.003
appTbSp (mm)	0.57 ± 0.07	< 0.001
appTbTh (mm)	− 0.23 ± 0.10	0.081
BMD (g/cm^2^)	– 0.12 ± 0.08	0.126

Model was adjusted for age, gender, body mass index, physical activity, smoking status, alcohol consumption, blood glucose, plasma glycated haemoglobin, lipids profile, and biochemical markers of bone metabolism.

App, apparent; BV/TV, bone volume/total volume fraction; TbTh, trabecular thickness; TbN, trabecular number; TbSp, trabecular separation; BMD, bone mineral density; SE, Standard error.

## 4 Discussion

Previous studies have shown that individuals with T1DM have a higher risk of fragility fractures compared to those without diabetes (1). However, our study did not find any differences in BMD at different bone sites between T1DM and non-diabetic controls, which is consistent with previous reports (22) and a meta-analysis (23). BMD alone does not fully capture bone strength, so assessing bone quality is important in understanding skeletal fragility in diabetes (24).

In our study, we utilized MRI to assess trabecular microarchitecture, specifically focusing on the parameters AppBV/TV, AppTb.N, AppTbTh, and AppTbS. We found that these parameters were altered in T1DM compared to controls, even after adjusting for various confounding factors. Similar findings have been reported in other studies using MRI and DXA to assess trabecular microarchitecture (15, 16, 25). One possible explanation for these alterations in bone quality in T1DM is the impact on bone marrow mesenchymal stem cells. Studies have indicated that diabetic bone marrow mesenchymal stem cells exhibit impaired ability to differentiate into osteoblasts, leading to increased expression of adipocyte markers and reduced osteoblast differentiation markers (26). Animal models of T1DM have also demonstrated an imbalance between bone and fat, with increased marrow fat accumulation and decreased bone mass. The receptor for advanced glycation end products may play a role in this process, as its chronic signaling in T1DM could disrupt bone marrow mesenchymal stromal cells and promote adipocyte differentiation (6, 11).

Historically, single-voxel MR spectroscopy with a single TE gained prominence for evaluating bone marrow fat fraction, particularly in the lumbar vertebrae region (27–30). owever, this method can yield biased results due to complexities such as overlapping water–fat peaks and differences in T2 relaxation times between water and fat signals (7, 20, 31–33). Trabecular bone presence reduces T2* relaxation time, exacerbating peak broadening and overlap among water–fat peaks. Furthermore, differing T2 relaxation times of water and fat introduce biases in fat fraction measurements, especially with a single TE (31). To address this, spectral data were collected at four TEs and meticulously corrected for T2 relaxation times, ensuring precise determination for both water and fat peaks. This correction, based on measured T2 values from multi-TE MR spectroscopy, effectively eliminates T2 weighting effects. Additionally, to mitigate J couplings impact, we employed a stimulated echo acquisition mode (STEAM) MR spectroscopy sequence with a short TE in this study (33, 34).

Consistent with previous findings, a recent study demonstrated the presence of an imbalance between bone and fat in the skeletons of mice with diabetes. This was characterized by an increase in fat accumulation within the bone marrow and a decrease in bone mass (2). The receptor for advanced glycation end products is believed to play a role in the development of fat tissue within the bone marrow. The disruption of bone marrow mesenchymal stromal cells in T1DM may be partly explained by chronic signaling of advanced glycation end products. However, when the receptor for advanced glycation end products was deleted, the up-regulation of factors that promote fat cell formation induced by T1DM in bone marrow stromal cells was prevented, as well as their differentiation into adipocytes (35). In T1DM mice, significant fatty deposits were observed within the marrow cavity of long bones. Notably, the levels of receptor activator of nuclear factor-kappaB ligand mRNA and protein were significantly increased in the adipose tissue of diabetic marrow, primarily located on the membranes of adipocytes (36). These findings suggest that increased bone resorption in early-stage T1DM mice is caused by receptor activator of nuclear factor-kappaB ligand derived from marrow adiposity rather than the bone tissue itself.

Several groups have investigated the impact of diabetes mellitus on bone marrow structures and functions in both human subjects and animal models. Animal studies consistently showed an increase in marrow adiposity (6, 11, 13, 37). However, the increase was not equal throughout the skeleton, with higher adiposity observed in diabetic calvaria and femurs compared to vertebrae (38). Human studies have shown mixed results, with some demonstrating higher marrow fat content in T1DM patients compared to controls (15, 16, 25), while others showed no significant difference (14, 17).

In contrast to previous human data, our study found a higher marrow fat fraction in T1DM patients compared to controls. Trabecular microstructures determined by MRI were identified as significant independent variables contributing to the marrow fat fraction. However, further studies are needed to fully understand this correlation. The conflicting results between animal models and patients may be due to confounding factors such as comorbidities and the limited size of clinical studies (39). Additionally, different measurement methods and specific analysis factors can also contribute to varying results.

Our study has some limitations. First, prior studies indicated that vertebral bone marrow heterogeneity seems to be primarily age-, sex- and menopause status-dependent (40, 41). Our study participants included males and females, both premenopausal and postmenopausal women. However, the sample size was not large enough to adequately compare different subgroups. Second, data on the severity of diabetes or other risk factors for bone deterioration were unavailable. Additionally, the cross-sectional survey design limits our ability to determine causality. Longitudinal studies would provide more insight into the progression of marrow adiposity in diabetes mellitus.

In conclusion, our study suggests that elevated marrow fat content in first-time diagnosed T1DM subjects may indicate altered trabecular bone microstructure associated with marrow adiposity. Incorporating marrow fat fraction measurement into MRI-based trabecular bone microarchitecture assessment could prove to be a valuable tool in the routine clinical evaluation of diabetic bone fragility.

## Data availability statement

The raw data supporting the conclusions of this article will be made available by the authors, without undue reservation.

## Ethics statement

The studies involving humans were approved by the Institutional Review Board of Shanghai University of Medicine & Health Sciences Affiliated Zhoupu Hospital. The studies were conducted in accordance with the local legislation and institutional requirements. The participants provided their written informed consent to participate in this study.

## Author contributions

WL: Methodology, Writing – original draft. WW: Data curation, Investigation, Writing – review & editing. MZ: Writing – review & editing, Formal analysis, Methodology. QC: Methodology, Writing – review & editing, Data curation. SL: Methodology, Writing – review & editing, Funding acquisition, Supervision, Validation.
